# Pregnancy, Birth, Neonatal, and Mental Health Outcomes Are Minimally Associated with Pregnancy Ambivalence

**DOI:** 10.1111/sifp.70045

**Published:** 2026-01-11

**Authors:** Karen Trister Grace, Samantha Auerbach, Amy Alspaugh, Nicholas Rios, Tara Altay, Samantha Kanselaar, Elizabeth A. Mosley

## Abstract

Pregnancy ambivalence is increasingly recognized and studied in sexual and reproductive health research, yet its associations with adverse outcomes remain unclear. The purpose of this paper was to explore different measures of ambivalence and whether any were associated with poor pregnancy, birth, social or mental health outcomes. A cross‐sectional survey was conducted with 1941 individuals assigned female at birth, ages 18–45, who had been pregnant in the past 2 years. Ambivalence measures included the London Measure of Unplanned Pregnancy (LMUP) and additional questions exploring mixed feelings, uncertainty, incongruent feelings, and fatalistic beliefs about pregnancy planning. No associations were observed between ambivalence and birth/neonatal outcomes, though ambivalence measures were linked to delayed prenatal care, exposure to harmful behaviors during pregnancy, and increased odds of depression, anxiety, and intimate partner violence. Mental health assessments and intimate partner violence screening could improve care delivery and outcomes more than screening for pregnancy ambivalence. The LMUP, which captures multiple dimensions of ambivalence as well as addresses the deficiencies with traditional measures of behavior, may be the strongest measure to use when needing to comprehensively measure pregnancy ambivalence.

## INTRODUCTION

Pregnancy intention is a complex, problematic, and controversial conceptual framework and construct. Often measured as a predictor of adverse health outcomes, pregnancy intention has been defined as how planned (Lyden et al. 2020) or intended (Kavanaugh and Schwarz 2009) a pregnancy was, with some measures encompassing wantedness (Phipps and Nunes 2012) and happiness (Rosengard et al. 2004) in reaction to the pregnancy as well. Barrett and colleagues developed a conceptual framework of pregnancy planning which consists of three broad dimensions: Context (encompassing circumstances and timing as well as partner agreement), Stance (encompassing desire, intentions, and feelings), and Behavior (encompassing planning and trying behaviors) (Barrett et al. 2004). Many researchers frame pregnancy intention as a binary concept (i.e., planned or unplanned, intended or unintended), with an assumption that planned and intended pregnancies are positive while unintended pregnancies are undesirable, and likely to be associated with negative outcomes. This approach has been criticized in recent years, due to the inconsistency of pregnancy planning with the lives and realities of some populations, who may not view pregnancy “planning” as a realistic or attainable goal, whose reproduction is devalued and stigmatized, or who may prefer pregnancy to be a “welcome surprise” (Dehlendorf et al. 2024; Edin and Kefalas 2005; Harris and Wolfe 2014). The public health goal of “planned” pregnancies has historically been exploited by racist and eugenicist movements that assign value to pregnancies among some populations (usually White, middle or upper class, able‐bodied, married, and heterosexual cisgender people), while devaluing pregnancies among others (S. L. Auerbach et al. 2023).

In recent years, a more nuanced approach to this complex construct has emerged that considers pregnancy intention as a continuum which includes the concept of ambivalence. Pregnancy ambivalence may be more salient to communities of color, lower income communities, and other marginalized groups, possibly reflecting cultural orientations toward pregnancy (views that pregnancy cannot or should not be planned, and will happen when meant to) (Borrero et al. 2015). Ambivalence may also reflect a reaction to discrimination against marginalized groups in society and healthcare, who are stigmatized by hegemonic ideals for “planned” pregnancy. There is little consensus on a definition or preferred measurement of ambivalence in the research literature. Some studies developed measures based on the dictionary definition of ambivalence (Gariepy et al. 2017; Lundsberg et al. 2020; Schünmann and Glasier 2006), which refers to mixed feelings which may be strong but contradictory (Merriam‐Webster Dictionary 2024). This corresponds to dimensions of ambivalence described in qualitative research, including incongruent responses, such as positive feelings about pregnancy while not taking steps to achieve or prevent it (Begun et al. 2019), not actively trying to get pregnant but feeling that it would not be a bad thing if it happened, having an unwanted pregnancy but not being upset about it due to having abortion access (Gómez et al. 2019), and paradoxical responses such as a strong desire to avoid pregnancy but also not using contraception (Kennedy et al. 2014). Other research defined pregnancy ambivalence as indifference (not caring (Suh et al. 2016; Xaverius et al. 2009) or having low levels of feelings on either side (Gariepy et al. 2017; Lundsberg et al. 2020)), uncertainty (Chuang et al. 2011; Gariepy et al. 2017; Jones 2017; Lundsberg et al. 2020; Mark and Cowan 2022; Quinn et al. 2021; Sable and Wilkinson 1998; Simmons et al. 2019), changing feelings (Gariepy et al. 2017; Kavanaugh and Schwarz 2009; Lundsberg et al. 2020; Schünmann and Glasier 2006), and lack of agreement with a partner (Kavanaugh and Schwarz 2009; Schünmann and Glasier 2006). Some continuous measures of pregnancy intention conceptualize ambivalence as existing between the extreme poles of wanted/intended and unwanted/unintended (Miller et al. 2008; O'Rourke et al. 2008; Phipps and Nunes 2012; Rosengard et al. 2004; Royer et al. 2016; Sable and Libbus 2000; Samari et al. 2020; Stulberg et al. 2020). Composite measures such as the London Measure of Unplanned Pregnancy (LMUP) encompass all of Barrett and colleagues’ dimensions of pregnancy planning, and suggest more nuanced categories that include ambivalence (Gariepy et al. 2017; Lundsberg et al. 2020; Schünmann and Glasier 2006).

Rates of pregnancy ambivalence vary widely, including 6.0% in population‐based US data from women with a recent pregnancy (Mohllajee et al. 2007), 36.4% of young adults in prospective population‐based US data (Higgins et al. 2012), and 38.1% of low‐income nonpregnant women seeking sexual and reproductive health services in Texas (Patel et al. 2015). Studies about the impact of unintended and unplanned pregnancy on adverse outcomes show conflicting results (i.e., large effects, small effects, and no effects), uncontrolled confounding factors, and significant risk for recall bias (Dehlendorf et al. 2024). Evidence of the impact of pregnancy ambivalence is equally unconvincing, and measures of ambivalence are found to frequently misclassify participant responses (Gómez et al. 2019). Some studies have found that adolescents and adults with symptoms of depression or stress were more likely to feel ambivalent about pregnancies (Francis et al. 2015; Patel et al. 2015), though it is unclear if ambivalence preceded depression or if depression led to ambivalent feelings about pregnancy. One study found ambivalence was significantly associated with increased odds of low birth weight (LBW; adjusted odds ratio [aOR] 1.15, 95% confidence interval [CI] 1.02–1.29) when compared to intended pregnancies; no other birth or maternal outcomes examined were significantly related (Mohllajee et al. 2007). Other studies have found that ambivalence toward future pregnancy was associated with less use of contraception (S. Auerbach et al. 2023; Yoo et al. 2014). Given the high prevalence of pregnancies categorized as “ambivalent,” more research is needed to examine whether pregnancy ambivalence is associated with poor outcomes for pregnant people and newborns.

Given the multiple ways pregnancy ambivalence is operationalized in the research literature, and the need to understand its possible association with maternal and newborn health outcomes, the purpose of this study was to explore multiple dimensions of pregnancy ambivalence measures and their associations with poor pregnancy, birth, social, and mental health outcomes.

## METHODS

We conducted a cross‐sectional survey with people assigned female at birth, ages 18–45, who had been pregnant in the past 2 years, regardless of pregnancy outcome, using the Prolific crowdsourcing platform. Prolific recruits diverse participants specifically for academic research studies, and is shown to have more reliable respondents and produce higher quality data than other crowdsourcing platforms (Douglas et al. 2023). Cognitive interviewing was conducted with three volunteers, who met eligibility criteria, to identify unexpected issues with wording and interpretation. Data were collected using REDCap between May and September 2023. Power analysis indicated a sample size of 2000 would be sufficient to detect statistically significant differences in key outcomes.

A total of 2715 people were invited to complete the survey, based on response to a preliminary screening question asking if they had been pregnant in the past 2 years, and 1998 completed the survey (74%). Forty‐eight people clicked “return” after completing surveys, indicating they had revoked their consent. To ensure data quality we included two attention check questions, each directing the participant to select a particular response if they were paying attention. Ninety‐one people failed one or both questions, and after reviewing these completed surveys for inconsistent responses or other suspicious signs, an additional nine survey respondents were removed from the sample. The final number of completed surveys was 1941—a completion rate of 71.5%.

The study was approved by the George Mason University Institutional Review Board (IRB 1985246‐1). Research assistants received standardized human subjects research ethics training as well as intimate partner violence (IPV) research training including safety assessment, technology safety, IPV resource referrals, and suicidality protocols. Participants reviewed tablet‐based survey/questionnaire informed consent covering the nature of the questions, information about confidentiality, and the voluntary nature of the study including that they can refuse to answer any question. Prolific encourages ethical reimbursement for research participation and suggests payment of $12 per hour. Anticipating the survey to take most participants 20–25 minutes to complete, we paid panel members $7.

### Measures

*Demographic measures* included age, income, sexual/gender identity, race/ethnicity, country of birth, education, family size, and relationship status.

*Discrimination* was measured with the nine‐item Everyday Discrimination Scale (EDS) (Krieger et al. 2005), which asks how often assorted experiences have occurred, and included items such as “You are treated with less courtesy than other people”; “people act as if they are afraid of you.” Responses ranged from 0 (Never) to 5 (Almost every day) and were summed (range 0–45). Participants were asked to choose the main reasons for these experiences from a list of 12 choices including “skin color,” “weight,” and “sexual orientation.” Internal consistency in our sample was excellent (Cronbach α = 0.91).

*Economic security* was measured with three items from the NISVS and BRFSS (2013, 2020). Items asked for frequency of worry or stress in the past 12 months about having enough money (1) to pay rent or mortgage; (2) to buy food for self and children (response options were always, usually, sometimes, rarely, or never, and were dichotomized into never or rarely and always, usually or sometimes); and (3) to see a doctor (yes/no).

Retrospective *pregnancy intention* was measured with the six‐item LMUP (continuous scale 0–12) (Barrett et al. 2004), which can also categorize pregnancies as planned (10–12), ambivalent (4–9), or unplanned (0–3). The LMUP is a rigorously developed measure which has good internal consistency and reliability (Cronbach α = 0.89 in our sample). Additional *de novo* questions measuring retrospective pregnancy Context, Stance, and Behavior were added to the survey to further explore dimensions of pregnancy ambivalence. Pregnancy ambivalence was operationalized as the following six responses:

- Context:
  ○ “I wasn't sure what I wanted” when asked about pregnancy timing (Centers for Disease Control and Prevention [CDC] 2016);
- Stance:
  ○ “Not sure” in response to “How did you feel when you found out you were pregnant with your new baby?” (Centers for Disease Control and Prevention [CDC] 2017);
  ○ “I had mixed feelings about having a baby” in response to “Just before I became pregnant…” (Barrett et al. 2004); and
- Behavior:
  ○ “Neither agree nor disagree” with the statements “I am trying very hard TO GET pregnant,” “I am NOT trying to get pregnant,” or “I am trying very hard to NOT get pregnant” (CDC 2017);

In an effort to capture incongruent responses, an additional dimension of ambivalence (Begun et al. 2019), we created an “incongruence” variable, which was operationalized as happy or very happy about the pregnancy *and* feeling the pregnancy happened at the wrong time, did not want to have a baby, or agree/strongly agree with “I am trying very hard NOT to get pregnant.” A additional measure, “Strongly agree” or “Agree” with “Pregnancy is not something you can plan, it just happens if it is meant to happen,” (CDC 2017) was also included. While not a measure of pregnancy ambivalence per se, this view often accompanies ambivalence (Jones 2018).

*Fatalism*, defined as feelings of luck, predetermination, and pessimism, was measured using a 20‐item scale (Shen et al. 2009) with responses from 1 (strongly disagree) to 5 (strongly agree) on questions including: “If someone was meant to get a serious disease, if doesn't matter what kinds of food they eat, they will get that disease anyway,” “I will die when I am fated to die,” and “Sometimes I feel that I am being pushed around in life.” Responses were averaged for a final score range of 1–5 with higher scores indicating a more fatalistic attitude. Internal consistency in our sample was excellent (Cronbach α = 0.90).

*IPV* was assessed with the Humiliation, Afraid, Rape, Kick (HARK) screening instrument, which measures experiences of emotional, sexual, and physical IPV (Sohal et al. 2007). Anyone who responded “yes” to any of the items was coded as having experienced any IPV in the past two years. Internal consistency (Cronbach α = 0.81) in our sample showed “good” reliability.

Questions about *pregnancy, birth and neonatal outcomes* were adapted from the Pregnancy Risk Assessment Monitoring System (PRAMS) measures (Centers for Disease Control and Prevention (CDC) 2024). Seven *pregnancy outcomes* were measured (late prenatal care, any smoke exposure in pregnancy, any substance use or alcohol use in pregnancy, gestational diabetes, vaginal bleeding, and severe nausea/vomiting/dehydration). Six *birth, postpartum, and neonatal outcomes* were measured (preterm birth, cesarean birth, induction of labor, prolonged newborn hospitalization, low birthweight, and any breastfeeding).

Five *mental health or social outcomes* were measured (antenatal, postpartum, and current depression/anxiety, and intimate partner violence). The Patient Health Questionnaire (PHQ‐9) was used to assess *depression* at the time of the survey, which is scored 0–27, and categorizes depression as minimal (1–4), mild (5–9), moderate (10–14), moderately severe (15–19), and severe (20–27) (Richardson et al. 2010). Internal consistency (Cronbach α = 0.92) was excellent in our sample. These categories were then dichotomized for analysis into none/minimal/mild and moderate to severe. *Anxiety* at the time of the survey was measured with the Overall Anxiety Severity and Impairment Scale (OASIS), a five‐item scale measuring anxiety severity and impairment, which is scored 0–25 and categorized as mild or none (0–5), moderate (6–10), severe (11–15), and extreme (16–25) (Norman et al. 2006). Internal consistency (Cronbach α = 0.93) in our sample was excellent. These categories were then dichotomized for analysis into mild or none and moderate to extreme.

### Statistical Analysis

Descriptive statistics (mean values, standard deviations, and frequencies) were used to describe the sample, the prevalence of pregnancy ambivalence measures, and the prevalence of outcomes. Chi‐square and *t*‐tests were used to examine differences between those who had pregnancy ambivalence with those who had intended/planned pregnancy or were happy about pregnancy, on risk factors and covariates. To account for possible confounding, variables that were significantly related (*p* < 0.05) to ambivalence in the univariate analyses were entered into an adjusted logistic regression model with each health outcome as the dependent variable. To avoid multicollinearity, we selected a single representative variable for duplicate or overlapping concepts such as rent/mortgage stress, financial stress, food stress, and education (income was used) and relationship status and marital status (relationship status was used). To account for inflated probabilities of false positives results (Type I errors) due to multiple testing, all the *p*‐values for the Wald tests for testing variable association with the health outcomes in the logistic regression models were adjusted using a Bonferroni correction. This keeps the probability of making a false positive discovery at 0.05 throughout the entire analysis. Mean imputation was conducted for one individual missing item in the pregnancy intention measure (*n* = 31 participants, 1.6% of full sample). Thereafter, the sample size floated to accommodate small amounts of missing data in other variables. Analyses were conducted in Stata (StataCorp 2025).

## RESULTS

### Sample Demographics, Pregnancy Ambivalence Measures, and Outcomes

The mean age of the sample was 31.0 (SD: 6.0, range: 18–45), and the most common pregnancy outcome was giving birth and parenting the baby (45.3%; Table 1). Over a quarter of participants (27.5%) identified as a sexual or gender minority, and the sample was evenly distributed across income levels, though around half reported experiencing rent/mortgage stress (55.7%) or food stress (47.8%). Approximately two‐thirds of the sample (67.1%) identified as White, 11.5% as multiracial, and 10.8% as Black, African American, or African. The majority of participants (81.5%) reported experiencing discrimination a few times a year or more, on the basis of skin color, gender, weight, or education/income. Of participants that identified as being Black, Hispanic, or multiracial, 39.3%, reported experiencing discrimination on the basis of race or skin color a few times a year or more (data not shown).

**TABLE 1:** Sample demographics, distribution of pregnancy ambivalence and pregnancy, birth, and mental health outcomes (*N* = 1941)

Variable	Mean (SD)/*N* (%)
*Age*	*31.02 (6.03)*
Result of the pregnancy	
Miscarriage or ectopic	523 (26.9%)
Abortion	305 (15.7%)
Stillborn (fetal demise)	15 (0.8%)
Gave birth/parenting	879 (45.3%)
Gave birth/someone else parenting	14 (0.7%)
Other	3 (0.2%)
Currently pregnant	202 (10.4%)
Sexual/gender minority	
Cis‐gender woman and heterosexual/straight	1405 (72.5%)
Sexual/gender minority	533 (27.5%)
Total 2022 household income before taxes	
$0–$25,000	225 (11.6%)
$25,001–$50,000	455 (23.4%)
$50,001–$75,000	472 (24.3%)
$75,001–$100,000	329 (17.0%)
$100,000 or more	436 (22.5%)
Unsure	24 (1.2%)
Rent/mortgage stress	
Never or rarely stressed about rent/mortgage	860 (44.3%)
Always, usually or sometimes stressed about rent/mortgage	1081 (55.7%)
Food stress	
Never or rarely stressed about food	1008 (52.2%)
Always, usually or sometimes stressed about food	922 (47.8%)
Needed to see a doctor but could not afford?	
No	1335 (68.8%)
Yes	606 (31.2%)
Race/ethnicity	
American Indian or Alaska Native	5 (0.3%)
Asian	68 (3.5%)
Black, African American, or African	210 (10.8%)
Hispanic, Latino, or Spanish	115 (5.9%)
Middle Eastern or North African	5 (0.3%)
Native Hawaiian or other Pacific Islander	3 (0.2%)
White	1302 (67.1%)
Multiracial	223 (11.5%)
Prefer not to answer	10 (0.5%)
Born in the United States	
No	69 (3.6%)
Yes	1872 (96.4%)
Education	
Some college or less	923 (47.6%)
College graduate or more	1018 (52.4%)
Family size (number of children under age 18 living with participant)	
0	545 (28.1%)
1	639 (32.9%)
2	466 (24.0%)
3	195 (10.0%)
4	63 (3.2%)
5	20 (1.0%)
More than 5	13 (0.7%)
Relationship status at time of pregnancy	
Not in a committed relationship	295 (15.2%)
In a committed relationship	1646 (84.8%)
*Mean fatalism score (range 1–5)*	*2.41 (0.62)*

The array of ambivalence measures yielded a wide range of responses (Table 2). The most commonly endorsed ambivalence measures were Stance measures: not sure how I felt when I found out I was pregnant (21.0%) and having mixed feelings about having a baby prior to getting pregnant (30.5%). Around one in 10 gave ambivalent responses when asked about Behavior measures (agreement with statements about how much they had been trying to get pregnant when the pregnancy occurred; 9.8%–12.5%). Around one in five participants gave ambivalent responses when asked the Context measure: feelings about pregnancy timing (18.5%). The least common measure of ambivalence was incongruence (5.7%). Around one in five (17.9%) also endorsed the view that pregnancy cannot be planned, but rather, just happens. Using the validated LMUP scale, 36.2% of participants were categorized as having pregnancy ambivalence.

**TABLE 2:** Univariate distribution of ambivalence predictors and outcomes (*N* = 1941 except where noted)

Variable	*N* (%)
	
**Pregnancy ambivalence dimensions and variables**	
Context	
Wasn't sure what I wanted (feelings about pregnancy timing)	360 (18.5%)
Stance	
Not sure how I felt when I found out I was pregnant	407 (21.0%)
Mixed feelings about having a baby, before I got pregnant	592 (30.5%)
Behavior	
Neither agree nor disagree with “I am trying very hard to GET pregnant”	196 (10.1%)
Neither agree nor disagree with “I am NOT trying to get pregnant”	191 (9.8%)
Neither agree nor disagree with “I am trying very hard NOT to get pregnant”	242 (12.5%)
Incongruence	111 (5.7%)
Agree/Strongly Agree with “Pregnancy is not something you can plan, it just happens”	347 (17.9%)
LMUP categories	
Planned	705 (36.3%)
Ambivalent	703 (36.2%)
Unplanned	533 (27.5%)
	
**Pregnancy‐related outcomes**	
Late or no prenatal care (*n* = 1111)^1^	80 (7.2%)
Any smoke exposure (from self or another person) in pregnancy (*n* = 1618)^2^	330 (20.4%)
Any drug use in pregnancy (*n* = 1636)^3^	353 (21.6%)
Any alcohol use in pregnancy (*n* = 1636)^4^	383 (23.4%)
Gestational diabetes (*n* = 11113)^1^	126 (11.3%)
Vaginal bleeding (*n* = 1418)^5^	259 (18.3%)
Severe nausea, vomiting, dehydration	394 (20.3%)
	
**Birth, postpartum, neonatal outcomes (*n* = 911)** ^6^	
Preterm delivery	75 (8.6%)
Method of delivery	
Vaginal	631 (70.1%)
Cesarean	269 (29.9%)
Labor induction	391 (43.8%)
Prolonged newborn hospitalization^7^	149 (16.6%)
Low birth weight (<5 lbs 8 oz)	64 (7.1%)
Any breastfeeding or pumping breastmilk	782 (87.3%)
	
**Mental health and social outcomes**	
Antenatal depression or anxiety	679 (35.0%)
Moderate, mod severe or severe depression at the time of the survey	641 (33.0%)
Postpartum depression or anxiety (*n* = 911)^6^	259 (28.4%)
Moderate, severe or extreme anxiety at time of survey	1162 (59.9%)
Any IPV in past 2 years	542 (28.5%)

^1^ Includes gave birth, stillborn, currently pregnant, excludes people who had abortions or miscarriages.

^2^ Any exposure to smoking in pregnancy from self or other person, excludes people who had abortions.

^3^ Any use of non‐OTC drugs during pregnancy, excludes people who had abortions.

^4^ Excludes people who had abortions.

^5^ Excludes people who had a miscarriage.

^6^ Includes only people who gave birth.

^7^ Six or more days for cesarean birth, 3 or more days for vaginal birth.

The most common prenatal outcomes were exposure to alcohol in pregnancy (23.4%), substance use (21.6%), and exposure to smoking (20.4%; Table 2). The most common birth‐related outcome was requiring labor induction (43.8%), followed by cesarean birth (29.9%). The majority of participants reported moderate to severe anxiety at the time of the survey (59.9%), and 33.0% reported depression.

In bivariate analysis (data not shown), socioeconomic status variables (income, rent stress, food stress, medical stress, and education), race, discrimination, relationship status, family size, age, and fatalism were each significantly associated with all measures of ambivalence except incongruent feelings, which was only associated with income, discrimination, relationship status, and fatalism. Participants with lower income or who identified as Black, Hispanic, or multiracial were more likely to have an ambivalent pregnancy (using the LMUP) and to provide ambivalent responses to other measures of preparation for and feelings about/reactions to pregnancy (*p* < 0.001 for all). Being born in the United States was only associated with one measure of ambivalence: uncertainty about pregnancy timing (*p* = 0.014). Significantly associated variables were controlled for in regression models (Table 3).

**TABLE 3:** Outcomes associated with pregnancy ambivalence when compared to pregnancies that were planned/happy/intended

	Measure of ambivalence (OR (95% CI) p‐value)^1^							
				*Behavior*				
	*Context*	*Stance*		Neither agree nor disagree with:				
Outcome	NOT SURE about timing	NOT SURE how I felt when I found out I was pregnant^2^	Had mixed feelings about having a baby, before I got pregnant^3^	“I am trying very hard to GET pregnant”	“I am NOT trying to get pregnant”	“I am trying very hard NOT to get pregnant”	Scored as Ambivalent on LMUP	Incongruence
Prenatal outcome								
**Late or no prenatal care (*n* = 1100** ^4^)	**4.24 (1.15, 15.65) 0.011**	2.26 (0.76, 6.71) 1.000	**3.39 (1.14, 10.07) 0.008**	2.63 (0.53, 12.96) 1.000	3.41 (0.84, 13.87) 0.262	1.53 (0.42, 5.56) 1.000	3.06 (0.91, 10.29) 0.147	1.20 (0.26, 5.57) 1.000
**Any smoke exposure in pregnancy (*n* = 1618** ^5^)	1.81 (0.96, 3.41) 0.126	1.27 (0.71, 2.29) 1.000	1.60 (0.92, 2.77) 0.351	1.19 (0.51, 2.81) 1.000	1.04 (0.45, 2.40) 1.000	0.90 (0.42, 1.90) 1.000	**2.03 (1.12, 3.67) 0.003**	1.44 (0.60, 3.42), 1.000
**Any substance use in pregnancy (*n* = 1636** ^6^)	**2.49 (1.33, 4.66) <0.001**	**2.12 (1.21, 3.73) <0.001**	**1.95 (1.13, 3.37) 0.002**	1.09 (0.46, 2.58) 1.000	1.27 (0.56, 2.89) 1.000	1.35 (0.68, 2.70) 1.000	**2.24 (1.24, 4.04) <0.001**	1.06 (0.43, 2.62) 1.000
**Any alcohol use in pregnancy (*n* = 1636** ^7^)	**2.27 (1.27, 4.08) <0.001**	**1.87 (1.10, 3.20) 0.004**	**1.68 (1.01, 2.79) 0.034**	1.37 (0.64, 2.97) 1.000	1.45 (0.70, 3.01) 1.000	1.25 (0.64, 2.43) 1.000	1.41 (0.84, 2.38) 1.000	0.92 (0.38, 2.22) 1.000
**Gestational Diabetes (*n* = 1113** ^4^)	0.95 (0.34, 2.69) 1.000	0.73 (0.26, 2.05) 1.000	1.17 (0.52, 2.63) 1.000	1.31 (0.44, 3.87) 1.000	2.07 (0.76, 5.66) 1.000	1.31 (0.47, 3.70) 1.000	1.23 (0.55, 2.71) 1.000	0.82 (0.19, 3.48) 1.000
**Vaginal bleeding (*n* = 1418** ^8^)	1.44 (0.68, 3.05) 1.000	1.36 (0.67, 2.75) 1.000	1.21 (0.64, 2.26) 1.000	0.77 (0.31, 1.94) 1.000	0.77 (0.30, 1.98) 1.000	1.59 (0.74, 3.44) 1.000	1.22 (0.65, 2.29) 1.000	0.62 (0.18, 2.12) 1.000
**Nausea/ vomiting/ dehydration (*n* = 1941)**	1.48 (0.83, 2.66) 1.000	1.38 (0.81, 1.85) 1.000	1.27 (0.76, 2.10) 1.000	0.82 (0.36, 2.26) 1.000	1.09 (0.51, 2.33) 1.000	1.14 (0.60, 2.17) 1.000	1.41 (0.84, 2.37) 1.000	1.27 (0.55, 2.91) 1.000

Birth, newborn, postpartum outcome^9^								
**Preterm birth (*n* = 860)**	1.53 (0.44, 5.35) 1.000	0.72 (0.19, 2.81) 1.000	0.96 (0.32, 2.90) 1.000	1.14 (0.28, 4.73) 1.000	1.35 (0.34, 5.34) 1.000	1.33 (0.37, 4.87) 1.000	1.09 (0.38, 3.12) 1.000	0.45 (0.05, 4.26) 1.000
**Cesarean birth (*n* = 900)**	1.10 (0.48, 2.56) 1.000	0.90 (0.41, 1.98) 1.000	0.86 (0.44, 1.69) 1.000	0.90 (0.36, 2.26) 1.000	0.88 (0.35, 2.23) 1.000	0.70 (0.28, 1.77) 1.000	0.89 (0.47, 1.69) 1.000	1.19 (0.41, 3.46) 1.000
**Induction of labor (*n* = 893)**	1.22 (0.56, 2.65) 1.000	0.97 (0.48, 1.97) 1.000	0.95 (0.51, 1.75) 1.000	1.07 (0.46, 2.50) 1.000	1.08 (0.47, 2.50) 1.000	1.22 (0.55, 2.69) 1.000	1.01 (0.57, 1.80) 1.000	1.29 (0.48, 3.47) 1.000
**Prolonged newborn hospitalization** ^10^ **(*n* = 898)**	0.95 (0.33, 2.73) 1.000	1.51 (0.62, 3.66) 1.000	1.09 (0.49, 2.44) 1.000	1.13 (0.38, 3.32) 1.000	0.85 (0.27, 2.66) 1.000	1.25 (0.46, 3.45) 1.000	0.99 (0.45, 2.17) 1.000	0.69 (0.16, 2.91) 1.000
**Low birthweight (*n* = 906)**	1.18 (0.30, 4.66) 1.000	0.61 (0.14, 2.60) 1.000	0.72 (0.22, 2.34) 1.000	1.79 (042, 7.62) 1.000	0.73 (0.14, 3.74) 1.000	0.35 (0.05, 2.17) 1.000	1.00 (0.33, 3.04) 1.000	1.00 (1.00, 1.00) 1.000
**Any breastfeeding (*n* = 896)**	0.59 (0.20, 1.77) 1.000	1.24 (0.41, 3.70) 1.000	0.85 (0.34, 2.14) 1.000	1.21 (0.30, 4.84) 1.000	1.44 (0.32, 6.38) 1.000	1.21 (0.32, 4.52) 1.000	0.66 (0.27, 1.62) 1.000	0.40 (0.12, 1.29) 0.767
**Mental health or social outcome**								
**Depression/anxiety in last pregnancy (*n* = 1941)**	1.39 (0.84, 2.31) 1.000	1.14 (0.71, 1.83) 1.000	1.29 (0.83, 2.00) 1.000	0.99 (0.51, 1.91) 1.000	0.97 (0.51, 1.85) 1.000	1.04 (0.59, 1.83) 1.000	1.26 (0.80, 1.97) 1.000	1.45 (0.69, 3.06) 1.000
**Depression at time of survey (*n* = 1941)**	**1.74 (1.01, 2.98) 0.036**	1.40 (0.85, 2.31) 1.000	1.48 (0.93, 2.36) 1.000	0.90 (0.44, 1.86) 1.000	1.06 (0.53, 2.15) 1.000	1.09 (0.59, 1.99) 1.000	1.54 (0.94, 2.51) 0.253	0.87 (0.39, 1.96) 1.000
**Postpartum depression (*n* = 911)** ^9^	1.12 (0.47, 2.64) 1.000	1.51 (0.71, 1.83) 1.000	1.26 (0.65, 2.46) 1.000	0.70 (0.26, 1.87) 1.000	0.81 (0.31, 2.15) 1.000	0.75 (0.29, 1.02) 1.000	1.10 (0.77, 1.57) 0.590	0.95 (0.32, 2.77) 1.000
**Anxiety at time of survey (*n* = 1941)**	**1.71 (1.02, 2.87) 0.026**	1.43 (0.89, 2.31) 1.000	**1.63 (1.06, 2.52) 0.008**	0.87 (0.46, 1.66) 1.000	1.04 (0.56, 1.95) 1.000	0.99 (0.56, 1.74) 1.000	1.54 (1.00, 2.38) 0.053	0.98 (0.45, 2.13) 1.000
**Any IPV (past 2 years) (*n* = 1902)**	**2.08 (1.18, 3.69) <0.001**	**2.00 (1.19, 3.36) <0.001**	**2.26 (1.38, 3.72) <0.001**	1.09 (0.47, 2.53) 1.000	1.92 (0.93, 3.96) 0.180	1.69 (0.92, 3.13) 0.317	**2.59 (1.50, 4.48) <0.001**	1.01 (0.44, 2.34) 1.000

^1^ All models control for income, race, relationship status, age, fatalism, sex/gender minority status, family size. All CIs and *p*‐values have been adjusted via the Bonferroni correction for the 162 hypothesis tests carried out in Tables 3 and 4.

^2^ Controls for all covariates, plus country of birth.

^3^ A component of the LMUP.

^4^ Includes gave birth, stillborn, currently pregnant, excludes people who had abortions or miscarriages.

^5^ Any exposure to smoking in pregnancy from self or other person, excludes people who had abortions.

^6^ Any use of non‐OTC drugs during pregnancy, excludes people who had abortions.

^7^ Excludes people who had abortions.

^8^ Excludes people who had miscarriages.

^9^ Includes only people who gave birth.

^10^ Six or more days for cesarean birth, 3 or more days for vaginal birth.

### Outcomes Associated with Pregnancy Ambivalence Measures

In multivariable analysis, no outcomes were significantly associated with any of the three Behavior measures. Three of seven prenatal outcomes were not significantly associated with any measure of ambivalence (gestational diabetes, vaginal bleeding, and nausea/vomiting/dehydration; Table 3). Four prenatal outcomes were positively associated with at least one ambivalent pregnancy measure (significant aORs range 1.68–4.24). Late prenatal care was positively associated with the Context measure and one Stance measure, substance use and alcohol use during pregnancy were each associated with all Context and Stance measures, smoking and substance use were associated with the composite LMUP measure of ambivalence. None of the birth/newborn/postpartum outcomes were significantly associated with any ambivalence measure. Of the mental health/social outcomes, antepartum depression/anxiety and postpartum depression were not significantly associated with any ambivalence measure. IPV was associated with all Context and Stance measures, as well as the composite LMUP (aOR range 2.00–2.59). Anxiety at the time of the survey was associated with the Context measure and one Stance measure (aOR range 1.63–1.71). And depression at the time of survey was associated with the Context measure (aOR 1.74, 95% CI 1.02–2.97).

The ambivalence measure associated with the greatest number of outcomes (six) was the Context measure, uncertainty about the timing of pregnancy (aOR range 1.71–4.24). Stance measures were associated with five different outcomes (aOR range 1.63–3.39). Scoring “ambivalent” on the LMUP was significantly associated with three outcomes (aOR 2.03–2.59). Incongruence was not significantly associated with any outcome.

### Fatalistic Views About Pregnancy Planning

Regarding agreement with the statement “Pregnancy is not something you can plan, it just happens,” 17.9% of the sample espoused this view (Table 2). In bivariate analyses (data not shown), all socioeconomic status variables except medical stress, as well as race, family size, and higher fatalism scores were significantly associated with this measure. Controlling for relevant demographic factors, multivariable regression results showed that four of the seven ambivalence measures were significantly associated with this fatalistic view (aORs range 1.91–2.51; Table 4), and notably, two of these were ambivalence on Behavior measures, which were not associated with any measured health outcome. In multivariable analyses, agreement with this fatalistic view was also not associated with any health outcome.

**TABLE 4:** Associations of measures of pregnancy ambivalence and health outcomes with fatalistic views about pregnancy planning

Outcome	Agree/Strongly Agree with “Pregnancy is not something you can plan, it just happens” (OR (95% CI) *p*‐value)^1^
**Ambivalence measure (*n* = 1941)**	
Context	
NOT SURE about timing	1.71 (0.96, 3.03) 0.117
Stance	
NOT SURE how I felt when I found out I was pregnant^2^	1.12 (0.65, 1.96) 1.000
Mixed feelings about having a baby, before I got pregnant^3^	**1.91 (1.18, 3.11) <0.001**
Behavior	
Neither agree nor disagree with:	
“I am trying very hard to GET pregnant”	**2.34 (1.16, 4.73) 0.002**
“I am NOT trying to get pregnant”	**2.51 (1.24, 5.08) <0.001**
“I am trying very hard NOT to get pregnant”	1.12 (0.56, 2.25) 1.000
Scored as Ambivalent on LMUP	**2.24 (1.38, 3.62) <0.001**
Incongruence	1.05 (0.40, 2.75) 1.000
**Health outcome**	
Late or no prenatal care (*n* = 1100)^4^	1.33 (0.45, 3.95) 1.000
Any smoke exposure in pregnancy (*n* = 1618)^5^	1.50 (0.83, 2.73) 1.000
Any substance use in pregnancy (*n* = 1636)^6^	1.19 (0.66, 2.17) 1.000
Any alcohol use in pregnancy (*n* = 1636)^7^	1.04 (0.59, 1.84) 1.000
Gestational Diabetes (*n* = 1113)^3^	1.13 (0.45, 2.87) 1.000
Vaginal bleeding (*n* = 1418)^8^	0.98 (0.48, 2.01) 1.000
Nausea/ vomiting/ dehydration (*n* = 1941)	1.39 (0.79, 2.43) 1.000
Preterm birth (*n* = 860)^9^	1.39 (0.45, 4.34) 1.000
Cesarean birth (*n* = 900)^9^	0.70 (0.33, 1.47) 1.000
Induction of labor (*n* = 893)^9^	1.30 (0.67, 2.55) 1.000
Prolonged newborn hospitalization (*n* = 898)^9^, ^10^	1.05 (0.44, 2.48) 1.000
Low birthweight (*n* = 906)^9^	2.09 (0.67, 6.53) 1.000
Any breastfeeding (*n* = 896)^9^	0.59 (0.23, 1.53) 1.000
Depression/anxiety in last pregnancy (*n* = 1941)	0.67 (0.40, 1.12) 0.868
Depression at time of survey (*n* = 1941)	0.78 (0.45, 1.32) 1.000
Postpartum depression (*n* = 911)^9^	0.71 (0.33, 1.51) 1.000
Anxiety at time of survey (*n* = 1941)	0.76 (0.46, 1.25) 1.000
Any IPV (past 2 years) (*n* = 1902)	1.03 (0.60, 1.77) 1.000

^1^ Controls for income, race, family size, fatalism, sexual/gender minority status. All CIs and *p*‐values have been adjusted via the Bonferroni correction for the 162 hypothesis tests carried out in Tables 3 and 4.

^2^ Controls for all covariates, plus country of birth.

^3^ A component of the LMUP.

^4^ Includes gave birth, stillborn, currently pregnant, excludes people who had abortions or miscarriages.

^5^ Any exposure to smoking in pregnancy from self or other person, excludes people who had abortions.

^6^ Any use of non‐OTC drugs during pregnancy, excludes people who had abortions.

^7^ Excludes people who had abortions.

^8^ Excludes people who had miscarriages.

^9^ Includes only people who gave birth.

^10^ Six or more days for cesarean birth, 3 or more days for vaginal birth.

### Sensitivity Analyses

To test the hypothesis that poor outcomes associated with pregnancy intention are actually due to confounding or mediating factors (Mohllajee et al. 2007), we re‐ran the regression models controlling for late prenatal care, substance use/smoking/alcohol use in pregnancy, and IPV in addition to the other covariates (data not shown). None of the previously significant associations remained. The *p*‐values for the sensitivity analyses were also Bonferroni adjusted to ensure that the probability of a false discovery was at most 0.05 within each sensitivity analysis.

Out of concern that our sample included people who were currently pregnant, who might be fundamentally different from participants who were recalling a past pregnancy, we re‐ran the regression models excluding these participants (data not shown). After doing this, three previously significant associations became nonsignificant (specifically, associations between the Context variable and depression at the time of the survey, and both Stance variables and alcohol use). Notably, one of the Behavior variables (I am not trying to get pregnant), which had no significant associations in our main analysis, became significant for four outcomes in this sensitivity analysis (smoking [*p* = 0.011], drug use [*p* < 0.001], alcohol use [*p* = 0.006], and IPV [*p* < 0.001]).

## DISCUSSION

This study reports findings from analysis of a large sample of people who were recently pregnant. Our sample was similar to national data on recently pregnant people, in key ways. In our sample, 7.2% of participants had late or no prenatal care, and 7.0% of women reported this across the United States (Osterman et al. 2025). About 29.9% of our sample had a cesarean birth, and this figure is 32.3% in national data (Osterman et al. 2025). Around 87.3% of our sample breastfed for any amount of time, compared to 91.2% in national data (Centers for Disease Control and Prevention [CDC] 2025). However, our sample was slightly older than the national average (31.0 in our sample, compared to 29.6) when using data on recent births (i.e., not including abortions and miscarriages) (Brown et al. 2025), and had a lower prevalence of preterm birth (8.6% in our sample, compared to 10.4% in national data) as well as a slightly lower prevalence of low birthweight newborns (7.1% in our sample, 8.6% nationally) (Osterman et al. 2025). Notably, our sample had much higher self‐reported rates of antenatal depression (35.0% compared to 16.8% in national data) and postpartum depression (28.4% compared to 12.7%) (CDC 2025). Population‐based data on pregnancy ambivalence is scarce, but one measure used by the Pregnancy Risk Assessment Monitoring System (“I wasn't sure what I wanted”) is comparable to our sample (15.5% compared to 18.5% of our sample endorsed this response) (CDC 2025).

The construct of pregnancy ambivalence is multi‐faceted, and it is measured in a variety of ways in the research literature. In this study, we used all available measures of ambivalence from existing research literature, as well as a measure of incongruent feelings, a composite measure, and a measure of a related construct often used in this research (pregnancy fatalism), to uncover associations with maternal and neonatal health outcomes. With the exception of incongruent feelings, all measures of pregnancy ambivalence were more likely to be endorsed by participants marginalized by race, ethnicity, and income. This may reflect that it is less acceptable for people in marginalized groups to report pregnancies as planned or intended, due to societal judgment of their being less worthy or capable of childbearing. Interpretation of these results requires grounding in the principles of Reproductive and Sexual Health Equity, a framework which centers marginalized communities, acknowledges past and ongoing harms, addresses root causes of health inequity, and honors bodily autonomy (Dehlendorf et al. 2021). This framework provides a lens through which to view pregnancy intention in relation to current and historical harms that restrict and judge reproduction by marginalized communities. The association between pregnancy ambivalence and race, racial discrimination and financial stress reflects structural drivers of ambivalence about pregnancy, not a lack of individual planning. Moreover, it is possible that experiences of marginalization could dampen or increase the effects of pregnancy ambivalence on perinatal outcomes.

Ambivalence measures that had the fewest associated outcomes were those that asked participants to recall levels of trying or not trying to get or not get pregnant (Behavior measures). The ambivalence measure with the most associated outcomes was the Context measure, which asked participants to recall feelings about the timing of the pregnancy. Stance measures (regarding feelings and reactions) and the composite LMUP scale had 3–5 significant outcomes associated. Notably, the LMUP includes items that ask about Stance, Context, and Behavior, but rather than ask about “trying” to get or not get pregnant it asks about objective indicators of these behaviors, such as regular use of contraception, taking folic acid, and seeking healthcare advice (Barrett et al. 2004). The construct of “trying” is a vague concept which may be difficult to recall, may be not meaningful to participants, or resonate with their experiences of becoming pregnant, and may be vulnerable to social desirability bias. A person who perceives their pregnancy to be stigmatized based on their age, race, income, or substance use, for example, may be less likely to report that they were trying to get pregnant, even if the pregnancy was highly desired. Asking participants to recall feelings and reactions may be more specific and easier to reliably measure. It is also possible that ambivalence about trying/avoiding pregnancy simply does not increase risk for poor outcomes. Existing studies of this construct are limited to outcomes of achieving pregnancy (Kavanaugh and Schwarz 2009; Wang et al. 2023) and use of emergency contraception (Royer et al. 2016).

For 12 of the 18 outcomes measured, including all birth/postpartum/newborn outcomes, there were no significant associations with any ambivalence measures, suggesting that the pathways linking ambivalence to outcomes may differ for behavioral versus physiological outcomes. Our results demonstrate consistently stronger associations (many were aOR 2.0 or higher) between measures of ambivalence and potentially damaging prenatal behaviors (smoking, substance use, late prenatal care) as well as depression or anxiety at the time of the survey and IPV. These behavioral and mental health outcomes are plausibly influenced by emotional and decision‐making pathways. For example, a person may delay prenatal care while deciding whether to continue a pregnancy they are unsure about, may be less able to prioritize pregnancy planning or to feel joy about a pregnancy if they have a substance use disorder or depression/anxiety, and may have mixed feelings about a pregnancy that occurs in an abusive relationship. In contrast, the lack of association between ambivalence and physiological outcomes (birth/postpartum/newborn outcomes) may reflect more indirect and biologically complex etiologies, such as chronic medical conditions and environmental exposures. Additionally, research has suggested that late prenatal care and substance use are potential confounders or mediators of the relationship between pregnancy ambivalence and other adverse outcomes (Mohllajee et al. 2007). Controlling for these and another plausible confounder (IPV) in sensitivity analysis explained all significant associations between ambivalence and adverse outcomes, which supports this hypothesis. Sensitivity analysis which excluded people who were currently pregnant revealed new significant associations with the Behavior ambivalence measure of “neither agree nor disagree” with “I am not trying to get pregnant.” It is possible that the double negative wording of this question was confusing to respondents and skewed the results, making this a spurious finding.

Other studies have found significant associations between ambivalence and some birth outcomes, such as LBW. One study found ambivalence was significantly associated with odds of LBW (aOR 1.15, 95% CI 1.02–1.29) when compared to intended pregnancies, but mistimed pregnancy had decreased odds of LBW (aOR 0.92, 95% CI 0.86–0.97) (Mohllajee et al. 2007). Researchers posit that statistical significance may not equate with clinical significance due to the weak magnitude of the ORs, and pregnancy intention is likely a marker for other factors like substance use and late prenatal care (Mohllajee et al. 2007). Unintended or unplanned pregnancy is more consistently documented with LBW in the literature; however, some researchers hypothesize this is due to confounding or mediation by other variables (Hall et al. 2017; Nelson et al. 2022; Shah et al. 2011).

Mood disorders were highly prevalent in our sample (one‐third reported depressive symptoms and almost 60% reported anxiety symptoms). Other studies have found similar associations between pregnancy ambivalence and depression (Francis et al. 2015; Lundsberg et al. 2020; Martínez‐Borba et al. 2020; Patel et al. 2015). One validation study of a Spanish pregnancy ambivalence scale used anxiety to test construct validity, due to the close correlation of these concepts (Martín‐Sánchez et al. 2022). Qualitative research has explored the close relationship between anxiety and ambivalence toward pregnancy and parenting, concluding that ambivalence is a normal and common experience, but can provoke severe guilt and fear, leading to anxiety (Folliard et al. 2024). In a society that expects women to joyfully and effortlessly experience pregnancy and parenting, it may be that ambivalent feelings are highly stressful and anxiety‐provoking.

The interpretation of ambivalence as feelings that are incongruent or paradoxical, such as trying not to get pregnant but feeling happy when it does occur was not significantly associated with any outcomes examined, nor was it significantly associated with race, with several socioeconomic variables, or with fatalism. One might assume that the stigmatization of pregnancy among marginalized people might increase the likelihood of feeling happiness about a pregnancy despite reporting other incongruent feelings or behaviors, but this did not appear to be the case in our sample. Limited research offers context for our findings. One study examined paradoxical combinations of intending pregnancy but using birth control, and not intending pregnancy but not using birth control, finding several significant associations with child health and development (Crissey 2005). Another examined incongruent combinations such as unwanted pregnancy and happy about the pregnancy, finding no significant association with plans for the future of the pregnancy or with contraceptive use (Sable and Libbus 2000). Qualitative research provides additional context, finding that incongruent feelings are not the same as ambivalent feelings, and that participants who expressed that pregnancy would be unacceptable while not using contraception, made clear unambiguous choices to terminate their pregnancies (Borrero et al. 2015).

Endorsement of the fatalistic sentiment that pregnancy cannot be planned—it “just happens”—was not significantly associated with any health outcome, but was significantly associated with several measures of pregnancy ambivalence, giving support to the argument that this sentiment is closely associated with pregnancy ambivalence (Jones 2018). This sentiment was significantly associated with races other than White and lower socioeconomic status in our sample. A qualitative study of low‐income African American and White women identified that life circumstances including a perception of reduced control over reproductive outcomes, feeling that financial stability before planning pregnancy was both necessary and unattainable, perceived sub‐fertility, and male partner reproductive coercion all contributed to this sentiment (Borrero et al. 2015). Other studies on the concept of pregnancy fatalism found mixed results depending on the definition of fatalism used. One study observed that endorsing a view that pregnancy is determined by fate was significantly associated with pregnancy ambivalence (defined as not seeking pregnancy but would feel okay if it happened) compared to not wanting pregnancy (aOR 1.83, 95% CI 1.05, 3.36, *p* ≤ 0.05), but endorsing the view that pregnancy “just happens” was not associated with ambivalence (Manze et al. 2021). Another study found that pregnancy ambivalence (defined as having unsure fertility intentions) was significantly associated with decreased odds of pregnancy fatalism (“It doesn't matter whether I use birth control, when it is my time to get pregnant, it will happen.”), compared to planning a future pregnancy (aOR 0.46, 95% CI 0.38, 0.56, *p* < 0.001) (Jones 2018). These constructs are complex and overlapping, and teasing out which components are implicated in poor outcomes is challenging. Although the current study did not assess interaction effects, it is possible that the relationship between pregnancy ambivalence (including fatalism) is moderated by people's social positionality and cultural background including their race, ethnicity, socioeconomic status, and experiences of discrimination.

The IPV literature identifies a clear association between IPV and measures of unintended pregnancy, which may be a result of specific IPV behaviors that focus on pregnancy promotion, called reproductive coercion (Grace and Anderson 2018). One meta‐analysis showed significantly increased risk of IPV with unintended pregnancy (aOR, 2.22, 95% CI 1.41–2.91) (Nelson et al. 2022). Studies found similar associations between IPV and pregnancy ambivalence (aOR range 1.53–2.27) (Patel et al. 2015; Robbins et al. 2021). This association doesn't necessarily mean that being ambivalent about pregnancy causes IPV; more likely, being in an abusive relationship causes very ambivalent feelings about pregnancy. Given the disproportionate impact of IPV on women (Black et al. 2011), driven by sexism and hypermasculine norms (Alonzo and Guerrero 2009; Casey et al. 2024), these results point to IPV as another socio‐structural driver of health inequity, in addition to racial discrimination and financial distress.

This study had several limitations which should be considered. This was a cross‐sectional survey, so conclusions cannot be drawn about causation. The study may be underpowered for rare binary outcomes such as LBW and preterm birth. All measures used were retrospective, which makes our findings vulnerable to the same reporting bias impacting other studies of pregnancy intention. We recommend future research addresses these questions using prospective design. Data for this study were collected from an online crowdsourcing platform, which carries risk of fraudulent responses. However, Prolific crowdsourcing platform recruits participants specifically for academic research studies, and is shown to produce high‐quality data (Douglas et al. 2023) and to be appropriate for surveys on sensitive topics such as violence, mental health, and substance use (Munro and Sellbom 2020; Stewart and Haselschwerdt 2023; Sahker et al. 2024). We utilized several recommended fraud reduction strategies, including spot checking for improbable answers and attention checks. Additionally, health outcomes were mostly self‐reported and vulnerable to participant misunderstanding or misremembering, which could under or overestimate their prevalence.

The sample was predominantly White, which is a limitation; however, the large sample size, sexual identity diversity, and the use of multiple measures of ambivalence are strengths of this study. Additionally, our sample was similar to national data on people who were recently pregnant. Future research should intentionally recruit to achieve racial and ethnic diversity in samples, and enable stratified analysis, to more closely align with the Reproductive & Sexual Health Equity framework at an earlier stage in the research process. Co‐development of new measures that are meaningful to marginalized and stigmatized communities and that predict outcomes identified by these communities as salient in the quest for health equity, is also a direction for future work. That pregnancy ambivalence reflects cultural and social constructs as well as stigma and discrimination, indicates the need for further exploration of the impact of socio‐structural factors on perinatal outcomes including how cultural background and social positionality might moderate the relationship between ambivalence and perinatal outcomes.

## CONCLUSIONS

This study highlights the complex and multifaceted nature of pregnancy ambivalence, examining the associations of multiple measures of pregnancy ambivalence with adverse perinatal, mental health, and social outcomes. No relationship emerged between ambivalence and birth or neonatal outcomes, but ambivalence was consistently associated with late prenatal care, smoke exposure, substance use, alcohol use, depression or anxiety at the time of the survey, and IPV. These findings suggest that measuring ambivalence through concrete, emotionally focused questions may better capture meaningful risk factors than the common questions about planning a pregnancy, which are ubiquitously used in clinical practice and research. The LMUP, which captures multiple dimensions of ambivalence as well as addresses the deficiencies with traditional measures of Behavior, may be the strongest measure to use when needing to comprehensively measure pregnancy ambivalence. The persistence of significant associations with mental health outcomes and IPV underscores the need for targeted interventions addressing these interconnected experiences. Focusing on ways to support individuals experiencing adverse mental health outcomes and IPV likely will have a more meaningful long‐term impact on their health and wellness than an intervention specific to pregnancy planning. This study also underscores the ways in which socio‐structural inequities—such as IPV, financial distress, and discrimination—contribute to experiences of pregnancy, including ambivalence. The lens of Reproductive and Sexual Health Equity further emphasizes the complicated ways in which reproduction can be constrained and stigmatized, reinforcing the role of sociocultural and policy changes needed to improve reproductive autonomy and health outcomes, thus moving us closer to the goal of health equity.

## Funding

This work was supported by the George Mason University, College of Public Health Pilot Funding.

## Conflicts of Interest Statement

KTG receives royalties from Wiley‐Blackwell publishers.

## Institutional Review Board Statement

The study was approved by the George Mason University Institutional Review Board (IRB 1985246‐1).

## Informed Consent Statement

Informed consent was obtained from all subjects involved in the study.

## Author Contributions

Conceptualization, KTG and EAM; methodology, KTG and TA; formal analysis, KTG, NR and SK; investigation, KTG and TA; data curation, KTG and SK; writing—original draft preparation, KTG, SA, AA, NR, and EAM; writing—review and editing, KTG, SA, AA, TA, SK, and EAM; visualization, KTG; supervision, KTG; project administration, KTG; funding acquisition, KTG. All authors have read and agreed to the published version of the manuscript.

## Data Availability

The data that support the findings of this study are available from the corresponding author upon reasonable request.
